# Co-creating a continuous leadership development program in rural municipal healthcare – an action research study

**DOI:** 10.1186/s12913-024-11096-8

**Published:** 2024-05-22

**Authors:** Trude Anita Hartviksen, Rita Solbakken, Lars Strauman, Inger-Lise Magnussen

**Affiliations:** 1 Center for Care Sciences, North, UiT, The Arctic University of Norway, PO Box 6050, Langnes, Tromsø, 9037 Norway; 2 Vestvågøy Municipality, Leknes, Norway; 3 Faculty of Nursing and Health Sciences, Nord University, Bodø, Norway; 4 Lofotleger AS, Leknes, Norway

**Keywords:** Action Research, Appreciative Inquiry, Leadership Development, Municipal Healthcare

## Abstract

**Background:**

An increasingly complex healthcare system entails an urgent need for competent and resilient leadership. However, there is a lack of extensive research on leadership development within healthcare. The knowledge gaps extend to various frameworks and contexts, particularly concerning municipal healthcare, knowledge leadership, and the application of knowledge in the field of practice. This study is the first in a larger action research project that aims to co-create a knowledge-based continuous leadership development program for healthcare in a rural Arctic municipality. This present study aims to explore the knowledge and experiences of the participating healthcare leaders to develop a common basis for co-creating the program.

**Methods:**

This hermeneutical study presents the first cycle of the larger action research project. An appreciative approach facilitated the project. Twenty-three healthcare leaders from three different leadership levels attended and evaluated two leadership development workshops and participated in four focus groups. The data were analyzed using Braun and Clarke’s reflexive thematic analysis.

**Results:**

Two main themes were identified: (1) changing from striving solo players to team players, and (2) learning to handle a conflicting and complex context. These results influenced how the leadership development program based on the participants’ co-creation was organized as a collective and relational process rather than an individual competence replenishment.

**Conclusions:**

The knowledge and experiences of healthcare leaders led to the co-creation of a knowledge-based continuous leadership development program based on the facilitated interaction of four essential elements: (1) competence development, (2) structures for interaction, (3) interpersonal safety, and (4) collective values and goals. The interaction was generated through trusted reflection facilitated by appreciative inquiry. The four elements and core played a crucial role in fostering relationships and facilitating learning, driving transformative change in this leadership development program. The study’s results provide a solid foundation for further co-creating the program. However, more research is needed to fully explore the practical application and overall significance.

**Supplementary Information:**

The online version contains supplementary material available at 10.1186/s12913-024-11096-8.

## Background

The healthcare system in the Western world is becoming increasingly complex, facing continuous development, changing expectations, new priorities, a growing number of older persons, and a significant shortage of human resources. To navigate these changes successfully, competent and resilient healthcare leadership is urgently required [1, 2]. However, healthcare leadership is recognized as demanding, characterized by high stress levels and a risk of burnout. The significance of leadership has often been undervalued, sometimes viewed merely as an adjunct to clinical tasks rather than as a vital component of system development [3].

### A knowledge-based leadership

Healthcare leadership is a decisive factor in ensuring that new knowledge, political decisions, and strategies are integrated into services that benefit individual citizens through up-to-date, knowledge-based practice. This has been referred to as “closing the quality chasm”. Additionally, leadership is pivotal in ensuring employee well-being, retention, and recruitment [3]. Healthcare leadership operates at several levels, described as the first-line, middle, and senior levels [4]. This study focuses on leadership development across all levels and various professions in municipal healthcare. However, these positions are primarily held by nurses, which is why the term “nursing leadership” is frequently used [5].

In a systematic review, Claesson et al. [6] elaborated on the unique and complex role of nursing leadership in municipal home-based healthcare. This role encompasses various elements, including trust and control, continuous learning, competence, skills, awareness of individual needs, mutual support and relationships, collaborative work at the organizational and interpersonal levels, nursing responsibility, and exposure to challenges. The review further concluded that nursing leaders must possess the qualities of multi-artists [6]. Jordal et al. [4] summarize how nursing leadership in municipal home-based healthcare entails professional, relational, financial, and organizational responsibilities as well as the demanding task of balancing multiple responsibilities [4]. There is a recognized need to clarify the leadership role in healthcare, establish boundaries around responsibilities, and enhance support from senior leaders [4, 5].

Knowledge management is presented as one of the answers to the changed needs in healthcare, understood as a systematic and organized approach to improve the organization’s ability to mobilize knowledge transfer. Although there has been limited research on knowledge management in healthcare [7], it is found to improve information and knowledge processes, decision-making capabilities, performance, quality of services, and to increase organizational effectiveness [8]. Knowledge leadership is presented as the most important key to succeed with knowledge management as it secures that the people involved are properly led, engaged, and motivated [9]. Knowledge leadership includes knowledge transfer, coaching, and the development of an operating culture for near future as well as long-term activity [7].

### Leadership development

Since the late 1980s, leadership development has evolved into an extensive industry and a burgeoning field of research, supported by an increasing array of theoretical frameworks [10, 11]. However, leadership development programs (LDPs) typically do not draw upon theoretical models or frameworks, as they are predominantly influenced by various competency models and team-based strategies [10]. Traditionally, there has been a greater emphasis on the leader’s individual development [12]. Leadership competence is identified as the knowledge, skills, behaviors, abilities, and attitudes that contribute to individual effectiveness [7]. While leader development focuses on intrapersonal growth, leadership development is recognized as interpersonal processes aimed at enhancing collective leadership capacity [10, 13]. A more collectivist approach to leadership development is suggested [13]. This knowledge is supported by the expectations of increased multi-professional collaboration and team-based municipal healthcare [2, 14]. Sørensen et al. [14] elaborate on the importance of enhancing organizational collaboration capabilities before introducing new professional teams, roles, and areas of responsibility. The key elements for strengthening such professional relationships are trust, respect, and continuity [14].

Even if theoretical frameworks employed in leadership development include learning by experience and interpersonal processes such as identity development [11], they have mainly centered on leadership styles [10, 11]. There are different understandings regarding which leadership styles are most suitable within healthcare. A collaborative leadership style is found conducive to knowledge management [9]. For leadership in contemporary healthcare, and as a method for enhancing leadership development strategies and outcomes, Alilyyani et al. [15] suggest an authentic leadership style, as delineated by Avolio et al. [16].

Several simultaneous learning processes are tested in leadership development, broadly categorized into two main forms: top-down versus bottom-up. Top-down processes encompass classroom lectures or reading texts about leadership, while bottom-up processes involve acquiring or enhancing leadership skills based on participants’ experiences. Some LDPs integrate these two forms of learning along with fundamental learning theories, leadership identity development, and motivation to lead and develop as a leader [10].

LDPs implemented in healthcare are typically adapted from other contexts [17]. The House of Leadership [18], presents a comprehensive leadership theory specifically developed for healthcare. This theory aims to facilitate the conversion of tacit knowledge into explicit knowledge and emphasizes the value of using organizational visions to guide leadership, and the necessity for leaders to engage in established networks. Hartviksen [19] outlines the need for a shift in the planning and execution of leadership development. This transition involves moving from unsupported to supported transformative processes, from healthcare leadership characterized by solitary competition to collaborative networks, and from a mission-based and controlling approach to empowering leadership. These changes encompass both pedagogical and relational principles and are suggested to draw from a broad theoretical foundation including complexity theory [20], learning theory [21], and leadership theory [22].

### Co-creation in healthcare research

Co-creation is depicted as crucial for the development of the public sector [23]. Greenhalgh et al. [24] describe co-creation in healthcare research as the collaborative generation of knowledge by academics working together with other stakeholders. They stress that a co-creative approach holds significant potential for social impact, contingent upon maintaining a systems perspective, adopting a creative approach to research focused on enhancing human experience, and giving careful attention to governance and process. Co-creation entails an interactive and dynamic process where value is created through the actual interaction that takes place [25].

Within healthcare, reference is made to a low scientific maturity regarding co-creative approaches [26]. Bowen et al. [27] describe how healthcare leaders encounter a top-down approach from researchers and decision-makers. Co-creative research has primarily presented influencing factors, with fewer studies presenting results [23]. Sharma [28] identified six characteristics of co-creative leadership: (1) creating a shared world view, (2) establishing a common vision, (3) fostering an environment of trust, (4) facilitating knowledge creation and sharing, (5) enhancing decision-making, and (6) promoting collaboration. Clausen et al. [29] explain that the pedagogical approaches guiding leadership development programs are as crucial as the program content itself. Reflection and narratives are highlighted as methods to address complex healthcare leadership challenges. Janamian et al. [30] found that a co-creative approach necessitates meaningful interactions, citizen-centered improvements, and co-creative governance, management, and communication within the research project. Openness, understanding, flexibility, fairness, and transparency are crucial [30].

There is a general need for further research in knowledge-based leadership development [7, 11], particularly across theoretical frameworks and concerning how knowledge is applied in the field of practice [11, 18]. Extensive research gaps have been identified from various perspectives both, related to municipal home-based healthcare leadership [4, 6] and healthcare leadership in general [7]. This study represents the first publication in a larger action research (AR) project aimed at co-creating a knowledge-based continuous LDP for healthcare in a rural Arctic municipality. It draws upon a theoretical foundation [20–22], as well as pedagogical and relational principles suggested by Hartviksen [19], to explore how these suggestions manifest in an active co-creation between professional practice and research [19]. The project comprises three consecutive studies with the same design. The subsequent study is planned to discuss pedagogical and relational methods, while the final study will present the ultimate results. This present study aims to explore the knowledge and experiences of participating healthcare leaders to develop a common basis for co-creating the program.

## Methods

This AR project originates from a broader reorganization process in a rural Arctic municipality in Norway involving comprehensive assessments to identify areas for improvement. The senior leaders requested that the authors of this study design a research project that would enhance leadership competencies, which was deemed crucial for cultural advancement. The study design is influenced by a hermeneutic stance [31] throughout all its research phases. All four authors have individual pre-understandings and have worked in various healthcare positions in rural Arctic municipalities, including leadership roles. TAH and LS have led a previous LDP that included this municipality and are currently employed there [32, 33]. RS participated in the previous program, and ILM has led other AR projects in rural Arctic municipal healthcare. As authors, we collectively recognize that leadership in municipal healthcare is diverse and often haphazardly achieved, and that it suffers from a lack of proper follow-up. Both TAH and RS have completed doctoral projects on healthcare leadership development [18, 19] and draw upon this knowledge in this present study.

### Design

**Fig. 1:**
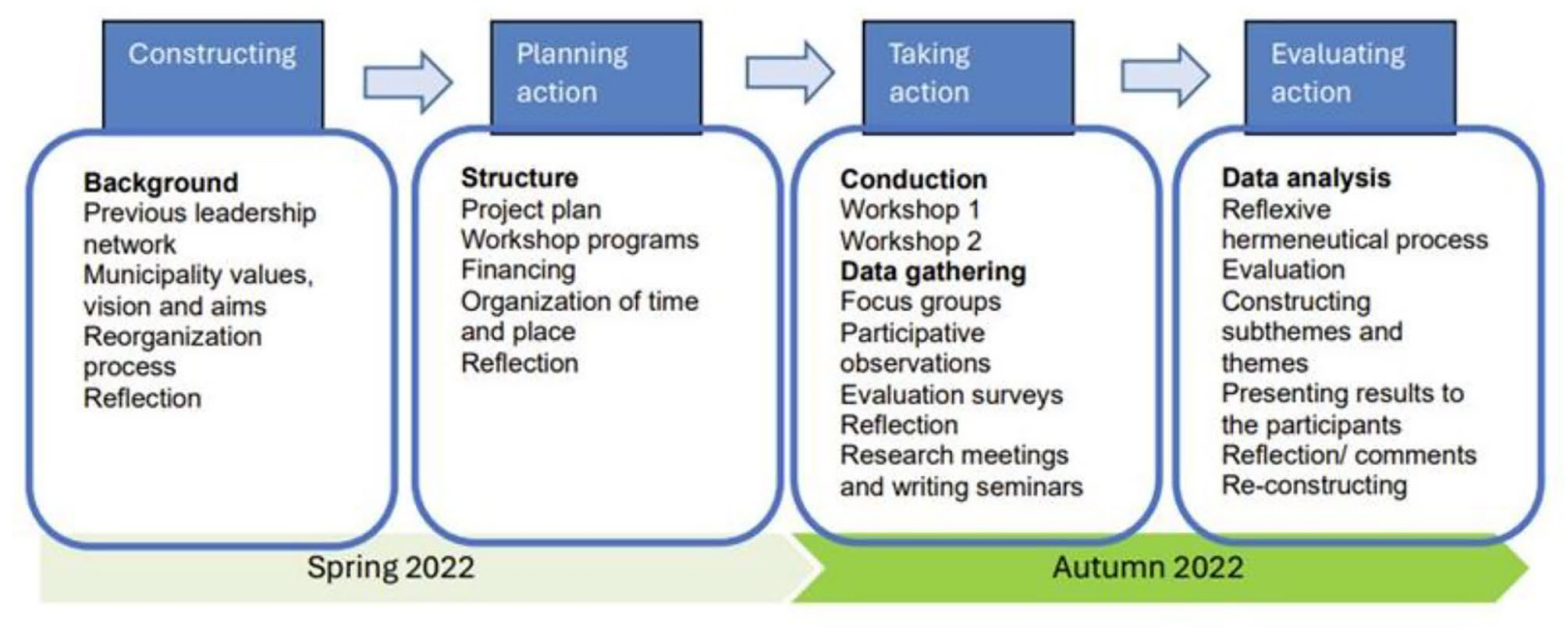
The action research process

Forming the initial part of a long-term AR project, the core elements of the design in this study are cyclical processes with co-creation [34–37] and appreciation [38]. Here, co-creation is understood as a “co-participation process” where we all create the organizational world to which we belong [39]. Appreciation is employed here as a philosophy, a manner of comprehending organizations, people, and the world, in a positive and future-oriented approach [38]. Aligned with the pragmatic nature of AR [40], and the capacity of appreciative inquiry to bridge positions and levels [41], we ensured co-creation from a bottom-up perspective through close collaborative interactions with the participants in the development, implementation, and evaluation of the research. The design followed four basic steps: constructing, planning, taking, and evaluating action; as visualized in Fig. 1.

The four steps in Fig. 1 were not rigid phases but overlapped and were continuously adapted to the situation and the participants, forming a flexible framework with reflections and revisions in a continuous back-and-forth process [31, 40]. An example of this process was demonstrated when the feedback from the participants in workshop 1 was systematically used to design workshop 2.

### Participants

This rural Arctic Norwegian municipality houses approximately 11,500 residents [42]. The municipal healthcare is organized with 10 units, 36 leaders, and around 500 employees. All leaders at three leadership levels in municipal healthcare were invited to participate—36 senior, middle, and first-line leaders in total. Of these, twenty-three leaders provided written informed consent to participate, with an emphasis on their right to withdraw at any point.

The leaders were not queried about their reasons for non-participation, yet several provided various explanations. Examples included extraordinary workplace situations requiring immediate attention, illness, and the observation that the focus group in the initial workshop was scheduled at the end of the first day, resulting in what participants deemed an excessively lengthy duration. Consequently, this was amended for the second workshop, resulting in the addition of three more participants. The leaders who did not participate held positions as first-line and middle leaders. Participation in the LDP was open to leaders irrespective of their involvement in the AR project. Further details regarding participant characteristics can be found in Table 1.

**Table 1:** Participant characteristics (*n* = 23)

Number	Profession	Further education	Leadership level	Age	Gender	Leadership experience
12	registered nurse (RN)	4 health leadership 1 intensive care 1 public health 1 geriatrics 1 gerontology and professional leadership 1 geriatric psychiatry and dementia 1 palliative care 1 personnel leadership and competence development 1 nutrition	6 first-line leaders 5 middle leaders 1 senior leader	37–63 (Mean = 47)	11 women 1 man	1–24 years (mean = 10.4)
1	RN and lawyer	none	1 senior leader	45	1 woman	1 year
1	child protection educator	pedagogical guidance	1 middle leader	55	1 woman	25 years
1	preschool teacher	health, environment, and security	1 first-line leader	55	1 woman	20 years
4	social educator	2 health leadership 1 coaching	3 first-line leaders 1 middle leader	36–58 (Mean = 46)	3 women 1 man	4.5–18 years (mean = 10.1)
1	social worker	public policy and administration health leadership	1 first-line leader	49	1 man	13 years
1	nurse assistant	none	1 first-line leader	61	1 woman	7 years
1	physician	personnel leadership and competence development	1 middle leader	34	1 woman	2 years
1	physio- therapist	none	1 first-line leader	32	1 woman	6 years
Total:						
23 participants	9 different professions	11 different further educations	3 leadership levels	32–61 (mean = 47)	20 women 3 men	1–25 years (mean = 10.4)

### Data gathering

The data were gathered through two workshops held in May and September 2022, each spanning two consecutive days at different conference venues. The methods included semi-structured focus groups (FGs), participatory observations, and online evaluation surveys. The workshop programs featured short introductory presentations on leadership-related topics, presented by the participants and the authors of this study. The programs facilitated reflection on the presented topics—individually, in groups, in the plenary, and after role-play. The accommodations, shared meals, intentional venue selection, and social activities aimed to foster interaction and professional networking [18].

Four FGs were moderated by TAH using semi-structured thematic interview guides (see Appendix 1 and 2) with open-ended questions [43]. The interview guides were prepared by all the authors based on previous knowledge and appreciative inquiry [44, 45]. Appreciative inquiry exerted a broad influence on the study design, notably on the interview guides and surveys, where we intentionally prioritized inquiries about strengths and positive attributes over weaknesses and negative qualities, as well as focusing on what enhances rather than what inhibits. This conversational technique was consistently employed in the researchers’ ongoing dialogue during the workshops, grounded in the philosophy of appreciation’s transformative potential for creation and change. It underscores the belief that language shapes the world we inhabit, encompassing opportunities and solutions, through its utilization [46].

Participants voluntarily formed two FGs in each workshop, consisting of seven to nine participants. RS and ILM co-moderated two FGs each, contributing additional questions to further explore the participants’ statements as well as handwritten notes describing the communication process and body language. The FGs were held in private rooms and were audio recorded; they lasted approximately one hour. A total of 30 h of participant observations by RS and ILM provided structured and unstructured field notes from the workshops, focusing on communication, commitment, body language, attitudes, roles, and interactions of the participating healthcare leaders [47].

Twenty-two and 19 participants provided anonymous feedback through online evaluation surveys (see Appendix 3) from the first and second workshops, respectively. These surveys, consisting of four questions on participant characteristics (position, experience, education, and leadership development) and an open-ended section asking to suggest improvements and topics that should be prioritized at the upcoming workshops, contributed to the rich empirical data analyzed in this study.

### Data analysis

A six-step reflexive thematic analysis [48, 49] was conducted as an active and recursive process between the parts and the whole of the empirical data [31] to explore latent meanings [50]. Reflexive thematic analysis is a theoretically flexible interpretative approach to qualitative data analysis that emphasizes the role of researchers [51]. All four authors engaged reflexively: (1) the data were read and re-read, (2) initial codes were generated, (3) potential themes were identified, (4) themes were developed through abstraction, (5) themes were defined and named, and (6) themes were communicated [48, 49].

The six-phase process was utilized as a set of guidelines rather than strict rules. Other researchers’ assessments and experiences of thematic analysis were of great help, particularly Byrne’s description, exemplification, and illustration of the analysis process of interview data in an educational context. Byrne’s [51] “worked example” served as a guide during four writing seminars with all authors present. During the comprehensive analysis process, the participants were actively engaged in providing input on the results. This involvement was facilitated through presentations of the results from the previous workshop and critical discussions held in the following workshop. These interactive sessions fostered productive discussions, ultimately enriching the depth of the analysis [52].

In the first phase, FG recordings were manually transcribed verbatim by TAH into 55 A4 pages of text. Handwritten notes and field notes were computer-written by RS and ILM, adding a total of 15 A4 pages to the empirical data along with 41 evaluation surveys. All four authors individually read and re-read the entire dataset, noting preliminary codes and themes (patterns of meaning). In phase two, ILM led a bottom-up approach to create concise codes, using color labels to examine both semantic and latent features. In phase three, the codes were clustered into six potential themes with substantiating subthemes [48, 49]. In phase four, all authors carefully assessed, abstracted, and refined the potential themes against the research question, codes, and subthemes [48]. It became necessary to return to the transcribed data and previous codes to ensure that no content was overlooked. The quality was ensured with the help of Braun & Clarke’s 15-point checklist [48]. This refining process led to four generated themes with distinct subthemes.

**Fig. 2:**
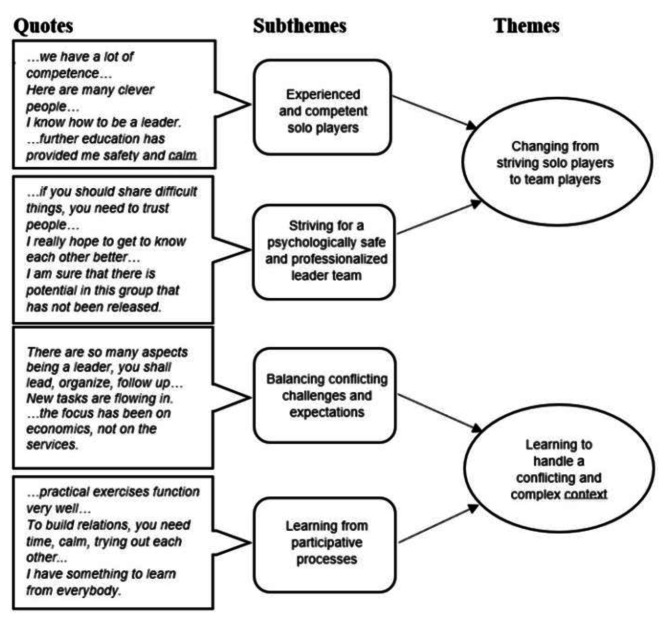
Themes and subthemes supported by quotes

In the fifth phase, each theme was related to the data and the research question one last time [48], requiring a deeper analysis of latent content [49]. Themes were refined, completed, and redefined by identifying their essence [48]. The four authors’ perspectives were discussed until a consensus was reached, developing a common horizon of understanding [31]. This process resulted in two final themes with substantiating subthemes. The sixth and final phase involved inspecting and organizing themes to form a logical, coherent structure [48]. The order of the themes illustrates how they relate to each other; quotes that support the themes and subthemes could first be related to the individual, second to the leadership team, and then to the complex context; as shown in Fig. 2.

## Results

Twenty-three healthcare leaders from a rural Arctic municipality participated in this study, including 12 registered nurses. The other 11 represented eight different health and social care professions. The participants reported 11 different types of further education. The ages of the participants ranged from 32 to 61 years, with a mean age of 47 years. Three men and 20 women participated, with leadership experience spanning one to 25 years (mean of 10.4 years). Participant characteristics are further detailed in Table 1. Two main themes were identified: (1) changing from striving solo players to team players, and (2) learning to handle a conflicting and complex context. Each main theme is substantiated by two subthemes.

### Changing from striving solo players to team players

This first main theme emerged from the results where the participating healthcare leaders described a deficit of trust within their organizational culture. Despite the participant characteristics demonstrating a highly competent and experienced leader group, the leaders acknowledged underutilizing their expertise across the municipal organization. Emphasizing the need for developing trust, participants advocated for a shift from individual-focused roles to a collaborative, team-oriented approach. This theme is substantiated by two subthemes: (1) experienced and competent solo players, and (2) striving for a psychologically safe and professionalized leader team.

### Experienced and competent solo players

In the FGs, participants across all three leadership levels conveyed fortuitous entries into leadership positions. Some had filled in for leaders on sick leave and later applied for the position, while others, with prior positions of trust, were encouraged to pursue leadership roles. Nevertheless, they explained having something valuable to contribute to leadership. The participants’ motivation for assuming leadership positions was frequently described as driven by a desire to influence development processes. They mainly depicted themselves as experienced leaders with further education. Even if the participants had participated in other LDPs, only one of them had experiences from the same LDP in which three of the authors of this study had been involved.

Both first-line and middle leaders described their leadership style as “present leadership,” emphasizing the importance of being accessible to employees. The first-line leaders in home-based services added to these descriptions when elaborating how they could be present for employees, even when they led from a distance most of the time. This was emphasized as being present when important, such as in the morning and before employees left work. Additional leadership styles, such as relational leadership and what participants referred to as “motherly leadership”, were identified; for example, as Participant 1 explained in FG 4:

*I said, hush, it must be quiet, and I must not hear it anymore, so, yes, so, it becomes a bit like that… educating, and maybe pointing fingers, but I think that’s when you must come up with that Health Personnel Act every now and then…*.

Despite their stated leadership competence, both first-line and middle leaders expressed a lack of confidence as leaders, thinking that the other leaders handled their jobs more professionally than they did themselves. They described feeling alone, lacking structured follow-up, and having inconsistent and short-term support for their leadership roles. Participant 9 explained in FG 2:

*… when we were to start up today, I just have to say that I do not have…. such enormous expectations for how this should be, because I am… terribly anxious that this will just be a blip, and then…*.

The middle leaders cited experiences of previous senior leaders having imposed sanctions when they disapproved of their actions, eroding trust, and fostering a more individualized leadership approach. Examples of such sanctions included publicly singling out individuals as negative examples, disregarding their contributions, or using suppressive techniques such as ridicule. As a result of these composite experiences, both the first-line and middle leaders described themselves more as solo players than team players.

### Striving for a psychologically safe and professionalized leader team

As part of the FG discussions, the participants emphasized a common interest in leading, achieving superior results, and working toward shared values and goals. However, they expressed a need for professional development as a leadership team, citing untapped potential due to a lack of collaboration across units. In both the second evaluation survey and the FGs, the participants underlined the need to develop safe relationships as essential for the LDP. This included cultivating positive leadership and ensuring stability within the leadership team. Changes in leadership positions had weakened the dynamics among them. However, the notes from the FGs and the participative observations described the participants as having active body language, listening to each other’s experiences, and asking follow-up questions.

It was discussed in the FGs that this municipal healthcare had been previously organized into two different areas, *health* and *caring*. This former organizational structure was occasionally visual. For example, in FG 4, Participant 2 expressed the following:

*I also miss being able to have more people from health involved in the leader meetings. Because those leader meetings are…very, very, useless for me, because the focus is on caring, and I am the only one from health who is present.*

​This middle leader described compensating for this by restoring a previous leader meeting, even though the participants in this meeting no longer held formal leadership positions.

While middle leaders described regularly meeting with each other, the overall leader group lacked shared meeting opportunities. Some first-line and middle leaders had initiated informal meetings to improve interaction. The participants suggested that the LDP should include cross-over groups to foster better understanding and interpersonal safety, as well as identify shared challenges. Some progress was made in this area at the end of the first workshop, as acknowledged by Participant 5 in the first FG:

*I think that I see that there is more trust; that is, in each other, we have become slightly more familiar with each other… there are quite a few constitutions and such, but maybe we are on to form a stable leadership group…*.

Some middle leaders described conflicts arising from previous financial discussions involving cutbacks, leading to competition over limited resources. This dynamic was experienced to hinder the ability to unleash their full potential. Despite this, participants recognized the positive aspects of differences in the leadership group, acknowledging the need to complement and learn from each other to develop as a professionalized leader team.

### Learning to handle a conflicting and complex context

The second main theme is developed from the results where the participants described their complex everyday lives with conflicting challenges and expectations. The healthcare leaders emphasized the need for a LDP that was strongly connected to these practical experiences and suggested participative learning methods as a means. This main theme is further substantiated by two subthemes: (1) balancing conflicting challenges and expectations, and (2) learning from participative processes.

### Balancing conflicting challenges and expectations

The participating first-line and middle leaders described in the FGs how the increasing complexity in municipal healthcare provided leadership challenges, additional tasks, and a larger total amount of work. Several first-line leaders shared how they found themselves in a challenging situation where they had to juggle dual roles 50/50 as both leaders and nurses in clinical work. They expressed difficulty maintaining satisfactory focus in either position, experiencing contradicting challenges and expectations. While the economic goals were described as “always clear,” they highlighted the absence of common goals and directions in other aspects, such as professional development. First-line and middle leaders described administrative tasks as “overwhelming.” They suggested that some of these tasks should have been done by others (e.g., human resources, office personnel), as they found it difficult to prioritize leadership tasks. For example, Participant 6 described in FG 3:

… *there can be a lot of administration and tasks meaning that you have little time to conduct quality work and improvement work…*.

The participants from all three leadership levels described how they assessed that different work requirements within the municipality healthcare increased complexity. They discussed this as creating a risk for unwanted variations in citizens’ quality of care and employees’ working conditions. An example was given where some employees worked every other weekend, while others worked every third weekend. The participants described several challenges within the municipal organization, relating most of these challenges to a need to improve the organizational culture. They described this as a time-consuming process tied to senior leadership levels, over which they had limited direct influence.

In the surveys, the participants exemplified challenges from their daily context. Here, feedback was given that they wanted to learn more about how to handle domination techniques, conflicts, difficult conversations, and employees in general. The participants requested to learn about how to motivate and create commitment and well-being when employees were tired and bored at work. Several first-line leaders explained in the FGs how they prioritized ensuring that the employees were well. Participant 8 in the fourth FG described this as follows:

…*if the employees are well, the citizens are well*…

Furthermore, in the surveys, the participants emphasized the need for additional knowledge about leadership, diverse leadership styles, leadership tools, long-term planning, involvement, and communication. Priority was expressed for fostering an environment of open expression and psychological safety, navigating change and processes, and implementing effective measurement strategies for improvement.

### Learning from participative processes

In the surveys, the participants described an overall satisfaction with the first workshop. In the second workshop, they were satisfied with the content and learning processes but not the venue. Criticism stemmed from its local setting, leading many to go home and forgo social events with dinner and quizzes. This was described as negative for developing social relationships and trust within the leadership team. The participation processes were highlighted as positive, especially regarding the FGs. This was exemplified when the moderator explained the time limit of the research project but clarified that the LDP was planned to continue independently of the research. One participant, supported by others, emphasized that the FGs needed to continue regardless, as they were crucial for their reflection. Additionally, participants provided positive feedback that preliminary results from the analysis were presented to them and noted that their input was incorporated into the further development of the LDP.

Feedback from the FGs emphasized the need for well-structured LDP sessions, incorporating frequent breaks. Survey insights recommended brief theoretical sessions balancing theory and group work. In the FGs, the participants emphasized that the topics should be narrowed down to what leaders found difficult or challenging. In both the FGs and surveys, the participants described having prior experience with personality tests, deeming them valuable for understanding diverse personality types within the leadership group.

According to the FG discussions, reflection was identified as a key component of leadership development, particularly in navigating demanding practical leadership situations. Participative observations confirmed the integration of reflection during the workshops, facilitated by the moderators’ open and appreciative inquiry. However, the participants lamented a minimal prioritization of reflection in their everyday routines. They suggested that LDP moderators took an active role in fostering reflection, in addition to how the program itself provided the necessary space, time, and distance from daily challenges. Participant 3 explained in the first FG:

*…if things are demanding, if you’re going to build relationships, then you need some time, you need calm, you need to brush up on each other a bit, be confident in each other, eh, daring to share difficult experiences from your own leadership.*

The desire for active and equal participation in the LDP was a unanimous sentiment across leadership levels in the FGs. Participant 11 in FG 2 elaborated:

*… we are all participants, but we must also contribute… I have the idea that I have something to learn from everyone, regardless of where and… what role my colleagues have or what kind of education we have. I think we are all equal…*.

Both the FGs and the survey feedback cited positive experiences with simulation and role-play, particularly in handling challenging situations. Unexpected incidents in the leaders’ daily roles revealed a lack of readiness, underscoring the importance of preparedness for unforeseen circumstances. However, despite the positive feedback, some leaders found role-play uncomfortable and emphasized the importance of ensuring that participants are not pressured into activities they are not adept at mastering.

## Discussion

**Fig. 3:**
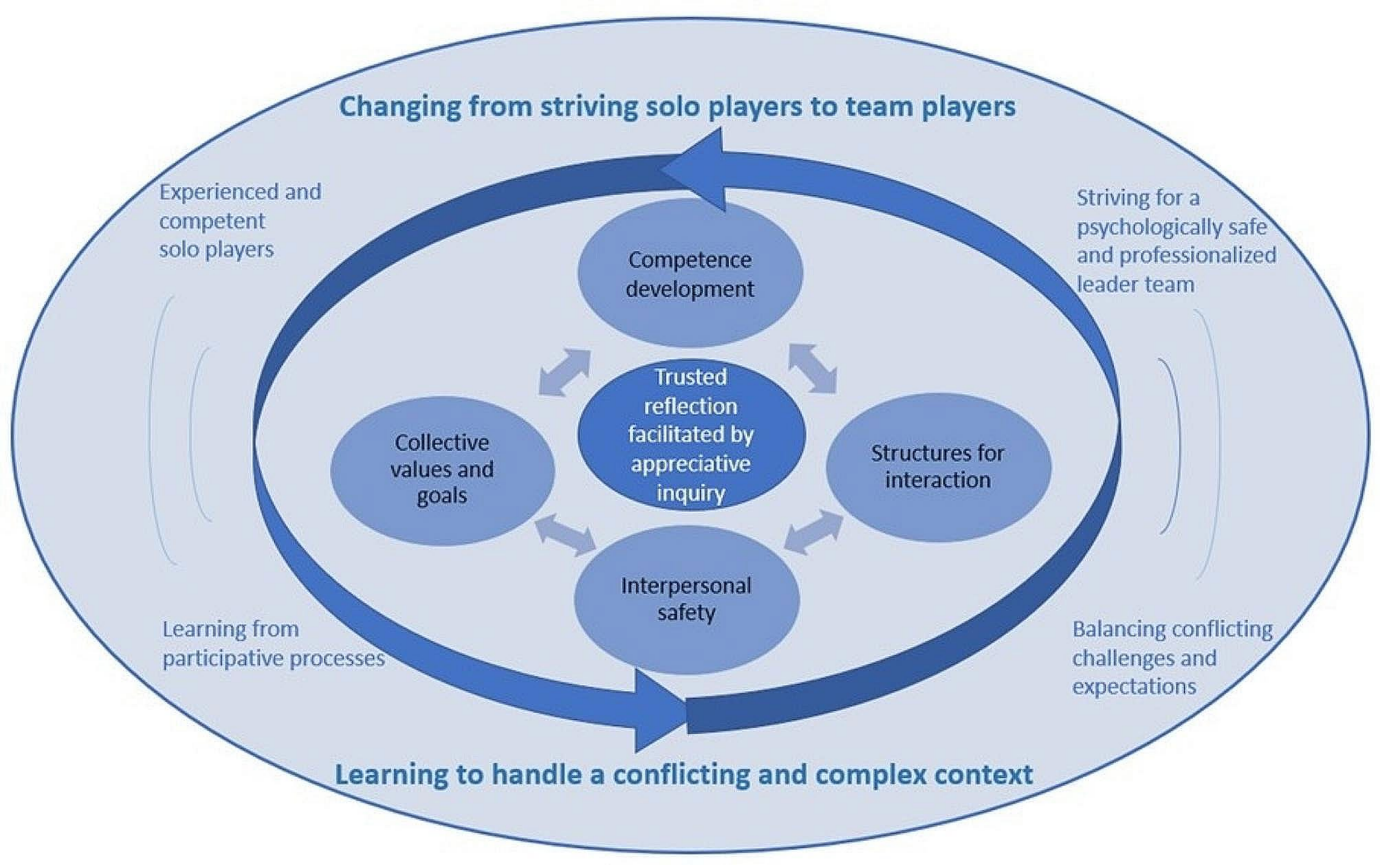
The appreciative co-creation process

This study explored the knowledge and experiences of healthcare leaders, developing a common basis for co-creating a knowledge-based continuous LDP in a rural Arctic municipality. Two main themes – *changing from striving solo players to team players* and *learning to handle a conflicting and complex context* – were delineated with substantiating subthemes. In our subsequent discussion, we will deliberate how the results contribute to existing knowledge, clarified within four essential elements: (1) competence development, (2) structures for interaction, (3) interpersonal safety, and (4) collective values and goals, and a core of trusted reflection facilitated by appreciative inquiry. The development of elements and core followed the hermeneutic process [31], merging insights from prior studies [18, 19] and the present study’s themes and subthemes, aligning complexity theory [20], learning theory [21], and leadership theory [22]. The elements and core, as illustrated in Fig. 3, will provide the common basis for the further co-creating of this knowledge-based continuous LDP. The relationship between themes, subthemes, elements and core will be further elaborated.

The core of Fig. 3, trusted reflection facilitated by appreciative inquiry, illustrates how, starting from the theoretical framework for the study [20–22] as well as the identified themes and sub-themes, this can be understood as the pedagogical and relational principles that grounded the process of developing this LDP. We have previously developed knowledge that identifies a need for transformative and continuous networks [18, 19] and LDPs [19] in the municipal healthcare. The core in Fig. 3 illustrates how this study adds to this knowledge by describing a potential driving force (engine) to ensure transformation and continuity. The themes and sub-themes in this study reveal, for example, a general lack of trust that prevents the collective utilization of leaders’ high individual competence for the overall good of the organization. For example, the results show how this lack of trust is reinforced by unwise former senior leadership, resource shortages and how preceding organizational structures still functioned as informal divisions of the municipal area. The core in Fig. 3 refers to how the design of this study, using appreciative inquiry, tailored an LDP that specifically addressed the participants’ needs. Emphasis was therefore placed on, through facilitated reflection, identifying trust-building factors and enabling redemption and utilization of each leader’s expertise in the group. This early insight influenced the selection of relevant topics as well as transformative relational [20] and pedagogical methods [21]. The core of Fig. 3 includes reliable reflective dialogue centered on listening to others’ perspectives, informed by hermeneutics [31] within the framework of the appreciative approach [44, 45].

Leadership competence has been identified as knowledge, skills, behaviors, abilities, and attitudes that contribute to individual effectiveness [7]. This study adds to this knowledge by illustrating the difference between developing individual and collective leadership competence. Competence development is thus one of the essential elements in Fig. 3. By emphasizing the relational importance of individual leaders’ articulation and further development of tacit knowledge, this knowledge contributes to The House of Leadership theory [18]. Mutual trust has been shown to have the potential to liberate people and their tacit knowledge [53]. Combining top-down and bottom-up learning processes [10], the latter was deliberately prioritized in this study in line with AR principles and the results that emerged. This included the results that substantiated the subtheme *learning from participative processes*, which pointed in the direction of transformative learning methods. The participants suggested several transformative pedagogical principles, such as learning through social interaction, critical reflection, open discourse, and the implementation of new understandings in practice [21]. Furthermore, the hermeneutic approach [31] implied a mutual development of competence through the co-creating process of the participants and the study authors.

Structures for interaction are an essential element in Fig. 3. This study developed knowledge about how the structured relational support offered in the LDP facilitated the results that emerged as themes and subthemes. Unlike other LDPs [10, 11], the present LDP does not focus on leadership styles. However, the results indicated that first-line and middle leaders experienced a transactional style from senior leadership. Although they described a more relational leadership style for themselves, which can be understood as transformative [16], they also referred to exercising a motherly leadership style. Based on their descriptions, this can be placed as transactional [16]. The first main theme, *changing from striving solo players to team players* with subthemes, thus suggests emphasizing a more transformative form of leadership in the LDP, which is known to support a more open and trust-based organizational culture [22]. Even if authentic leadership is described as including relational transparency [16], this knowledge is further developed within this study, highlighting structures for interaction as the starting point for developing collective leadership competence. The LDP was offered to leaders from all three leadership levels in the municipal area. This strengthened first-line and middle leaders’ opportunities for support and interaction with senior leaders, which is a known weakness in municipal healthcare [13].

Interpersonal safety, as an essential element in Fig. 3, refers to how the results from this study differ from the knowledge provided by other LDPs that focus on individual leadership competence or the group’s leadership ability [10]. The co-creating process in this study altered the focus from individual development to working on the relationships between the leaders. This supports the knowledge about the benefits of a more collectivist approach to leadership [2, 10, 12, 14], and how trust, respect, and continuity are suggested to improve professional relationships [14]. The participants showed interest in each other’s experiences, listened, and asked follow-up questions. This was interpreted as a sign of developing trust and interpersonal safety. Informal meeting points, such as shared meals, accommodations, and social activities were a key part of building interpersonal safety; understood as getting to know each other as people, not just colleagues, challenging each other and laughing together.

Collective values and goals that are superior to leaders’ individual priorities [44] are the fourth essential element in Fig. 3. Despite how the participants’ individual values and goals of being present and visible leaders were clarified through the results of this study, this element refers to how they were unsure of the municipality’s shared values and goals, felt alone, and experienced a lack of support and follow-up. These results support existing knowledge describing a need to establish boundaries around the responsibility of healthcare leadership and increase the support from senior leaders [13]. This study adds to this knowledge by identifying the vulnerability of these deficiencies to healthcare organizations, and describing how they reduce leaders’ ability to prioritize leadership, development-related tasks, and quality improvement. The results showed that only one of the participants in this study had participated in the previous LDP in which three of the authors had been involved, which suggests a high turnover within the leadership group.

Overall, the results from this study support the descriptions of increasing complexity and continuous change in healthcare [1, 2], and the understanding of leadership as a dynamic process based on interaction [20]. As such, municipal healthcare can be understood as a mesosystem where the LDP becomes a temporary relational process that is difficult to construct in advance. The non-linear nature of the interaction means that one needs to try out the methods and evaluate the results it produces, then make the necessary changes [20]. This knowledge implies that the construction of this continuous LDP must be subject to ongoing development, trying to accommodate the constantly changing contextual conditions. The arrows and movement in Fig. 3 indicate how the synergies between the elements bring the LDP forward, supported by the research phases in the hermeneutical AR approach [31, 40]. The four elements mutually influence one another and the core. Thus, it is reasonable to think that neglect or challenges in any of the elements may disrupt continuity, hindering results similar to the themes and subthemes presented in this study.

The results support The House of Leadership theory [18], depicting that when participation in formal networks is not offered, informal networks arise within the organization. However, we have not considered the significance for the field of practice within this study [11, 18]. This will need further research. The results provide an example of how fundamental changes after reorganization are challenged when the underlying patterns in the system are not challenged and/or changed and when the ground is not prepared for upcoming changes [1, 2]. The necessity of this continuous LDP was further emphasized by the leaders’ statements that this was the only meeting point of its kind in the organization.

### Strengths and limitations

Despite how previous studies have acknowledged the methodological advantages of co-creation [12, 29], we have not found studies that have used AR to co-create LDPs [1, 6, 7, 12, 13, 15]. The use of AR offers several strengths to this study. First, the research was initiated from a shared concern among researchers and participants. Second, “inside information” was secured when only participants who worked within the practice of concern participated. Third, the methods involved continuous adjustments based on participant feedback, fostering an adaptive approach aligned with the dynamic nature of the practice [40, 54]. Knowledge contributed from a previous LDP in which the municipality participated prepared the stage with shared visions [32, 33]. Thus, the study could be described as building on the past and taking place in the present with a view to shaping the future [40, 55]. Member checking, also known as “participant validation,” was used as a technique for exploring the credibility of results [52]. The interplay of action and research, characteristic of the dynamic nature of AR, provided a platform for collaboratively co-developing, theorizing, testing, and implementing knowledge within an evolving leadership landscape [37].

Triangulation of the methods may have provided deeper meaning to the results [37] and helped minimize the limitations of each method [56]. The use of mixed groups of participants in the FGs, with diverse competencies, experiences, and skills enriched the empirical data and added diverse leadership perspectives. However, this could also provide limitations wherein leaders across various hierarchies engage in the same FG. Divergent power dynamics might lead participants to tailor their contributions to accommodate the presence of leaders either above or below their own rank. Contextual factors such as the rural setting could also have further shaped the dialogue, potentially due to pre-existing relationships among participants or their familiarity outside the formal setting. The co-moderators diligently examined these communication dynamics within the FGs but did not highlight any such observations in the field notes. The overall data contributed to topics and situations being illuminated and reflected upon from several perspectives, strengthening the trustworthiness of the results [40].

Although the six analysis steps were indicative, this reflexive approach safeguarded the flexibility and openness of the analysis and is known to strengthen credibility and trustworthiness. However, a more theoretical driven analytic framework might have brought in other perspectives. The reflexivity of the analysis was strengthened by the fact that all the authors brought their perspectives into the analysis and that for one of them, both the participants and the context were unknown [49].

AR is criticized for how the researcher’s proximity to participants and the research field may “color” actions and data. Given the possible pitfalls of the analysis [48], we have described each phase in detail to ensure that the data are processed respectfully. In the context of this limited research community, the act of sharing experiences may pose challenges to maintaining anonymity and confidentiality [57]. To mitigate potential issues and maintain transparency, we proactively fostered an open dialogue with the participants throughout the study [37]. AR provides the researcher with a unique opportunity to observe, discuss, and reflect upon the participants, which contributes to the trustworthiness and credibility of the results. The participants’ openness and willingness to engage strengthened the study. Ensuring equality was a fundamental principle from the researchers’ standpoint, aligned with the core characteristic of AR [57]. Every participant’s contribution and role were accorded equal values, emphasizing the democratic nature of the collaborative process. The study’s commitment to fostering knowledge and practice development underscores the transformative potential of AR and its cooperative essence rooted in a bottom-up perspective [37].

## Conclusions

Healthcare leaders in a rural Arctic municipality provided valuable knowledge and experience when co-creating a continuous LDP. This insight demonstrated each leader’s high level of competence, while overall organizational competence was nonetheless considered low. This contrast was found to be due to lower levels of trust and interaction. The results were delineated into two main themes with substantiating subthemes: (1) changing from striving solo players to team players; and (2) learning to handle a conflicting and complex context. Based on existing knowledge, the results from this study, and relevant theoretical perspectives, four essential elements were clarified in the co-creation of the LDP. These elements were: (1) competence development; (2) structures for collaboration; (3) interpersonal safety; and (4) collective values and goals. The core of these elements was identified as trusted reflection facilitated by appreciative inquiry. The elements are understood as instrumental to the process of transformative change in this LDP, having a pivotal role in fostering relationship building and facilitating learning. Thus, the result of this study and the derived essential elements are considered a solid starting point for further co-creation of the LDP. This study contributes to the research field with knowledge of how an appreciative co-creation process can build results based on the leaders’ knowledge and experiences in municipal healthcare. However, the study’s main contribution is the suggestion of four essential elements that can act as a common basis for municipal healthcare in planning and implementing a knowledge-based continuous LDP. In terms of society, knowledge of how to strengthen and develop leadership will be crucial to carry out the necessary changes required to safeguard municipal healthcare in a long-term perspective. Nevertheless, in-depth research is imperative in order to comprehensively explore the practical applications and overall significance of the results presented.

### Electronic supplementary material

Below is the link to the electronic supplementary material.

Supplementary Material 1

Supplementary Material 2

Supplementary Material 3

## Data Availability

The datasets generated and analyzed during the present study are not publicly available due to the confidentiality afforded study participants but are available from the corresponding author upon reasonable request.

## Abbreviations

AR: Action Research
FG: Focus Group
LDP: Leadership Development Program
