# Supplementary material for: Co-creating a continuous leadership development program in rural municipal healthcare – an action research study
Source: BMC Health Serv Res. 2024 May 22;24:656. doi: 10.1186/s12913-024-11096-8 (PMC11112788; doi:10.1186/s12913-024-11096-8)
Supplement: Supplementary file 1 — Supplementary Material 1 [file 12913_2024_11096_MOESM1_ESM.pdf]

Appendix 1, 1. Focus group discussion guide

Aim: to explore the knowledge and experiences of healthcare leaders, developing a common basis for co-creating a knowledge-based continuous leadership development program in a rural Arctic municipality.

Background for the question	Research question	Question to the focus group discussion guide
Introductory questions	Participant characteristics	How old are you?
		What basic education do you have?
		Do you have further education?
		How long have you been a leader?
		What inspired you to become a leader?
		Has this inspiration changed?
		Have you participated in leadership development before?
Mapping questions	How is leadership understood?	What is leadership for you?
		How would you describe the exercise of leadership in your leadership group?
		What do you think promote the opportunity to exercise leadership as you wish?
		What do you think inhibit the ability to exercise leadership as you wish?
		What are this leadership group's strengths?
		What are this leadership group's weaknesses?
	Which topics are relevant to include in a continuous leadership development program?	What do you lack knowledge about to exercise leadership in such a way that you experience mastering this role?
	How should a leadership development program be designed to provide the best possible benefit for the participant?	How do you think a continuous leadership development program should be carried out in order for you to experience the best possible development, for example in relation to lectures, group work, individual work?
		How often should we meet in the leadership development program?
		What length of meetings do you think will work best in the leadership development program?
Follow-up questions	How can we gain more in-depth knowledge within the various parts of the research project?	Can you provide more examples?
		Can you elaborate more?
		Does anyone have anything to add?
		How did this happen?
		How do you know this?
		What gave good results or bad results as you see it?
		Have you thought about how this could be improved?
