# Supplementary material for: Co-creating a continuous leadership development program in rural municipal healthcare – an action research study
Source: BMC Health Serv Res. 2024 May 22;24:656. doi: 10.1186/s12913-024-11096-8 (PMC11112788; doi:10.1186/s12913-024-11096-8)
Supplement: Supplementary file 2 — Supplementary Material 2 [file 12913_2024_11096_MOESM2_ESM.pdf]

## Appendix 2, 2. Focus group discussion guide

Aim: to explore the knowledge and experiences of healthcare leaders, developing a common basis for co-creating a knowledge-based continuous leadership development program in a rural Arctic municipality.

| Background for the question | Research question | Question to the focus group discussion guide |
|---|---|---|
| Introductory questions | Participant characteristics | How old are you? |
| | | What basic education do you have? |
| | | Do you have further education? |
| | | How long have you been a leader? |
| | | What inspired you to become a leader? |
| | | Has this inspiration changed? |
| | | Have you participated in leadership development before? |
| Mapping questions | How do you think your personal characteristics affect your leadership style? | Who are you as a leader? What are your characteristics? |
| | | How is the leadership group affected by the various leader characteristics? |
| | | What do you think promote the opportunity to exercise leadership as you wish? |
| | | What do you think inhibit the ability to exercise leadership as you wish? |
| | | What are this leadership group's strengths? |
| | | What are this leadership group's weaknesses? |
| | | Have there been any changes in the leadership group since the first leadership development program? |
| | Which topics are relevant to include in a continuous leadership development program? | What do you lack knowledge about to exercise leadership in such a way that you experience mastering this role? |
| | How should a leadership development program be designed to provide the best possible benefit for the participant? | How do you think a continuous leadership development program should be carried out in order for you to experience the best possible development, for example in relation to lectures, group work, individual work? |
| | | How often should we meet in the leadership development program? |
| | | What length of meetings do you think will work best in the leadership development program? |
| Follow-up questions | How can we gain more in-depth knowledge within the various parts of the research project? | Can you provide more examples? |
| | | Can you elaborate more? |
| | | Does anyone have anything to add? |
| | | How did this happen? |
| | | How do you know this? |
| | | What gave good results or bad results as you see it? |
| | | Have you thought about how this could be improved? |
