# Supplementary material for: Co-creating a continuous leadership development program in rural municipal healthcare – an action research study
Source: BMC Health Serv Res. 2024 May 22;24:656. doi: 10.1186/s12913-024-11096-8 (PMC11112788; doi:10.1186/s12913-024-11096-8)
Supplement: Supplementary file 3 — Supplementary Material 3 [file 12913_2024_11096_MOESM3_ESM.pdf]

Evaluation survey

Continuous leadership development program

1. What leader position do you have?

- ☐ First-line leader
- ☐ Middel leader
- ☐ Senior leader

2. How long have you been a leader?

- ☐ 0-2 years
- ☐ 2-5 years
- ☐ 5-10 years
- ☐ More than 10 years

3. Have you participated in other leadership development previously?

- ☐ Yes
- ☐ No

4. If you have participated in other leadership development previously, which?

- ☐ Courses organized by the municipality
- ☐ Courses organized by others
- ☐ Further education
- ☐ Other

5. Do you have comments, suggestions for improvements or current topics that you would like us to prioritize in the future?

____

____

____
